# Genetic Landscape of Obesity in Children: Research Advances and Prospects

**DOI:** 10.1155/jobe/9186826

**Published:** 2025-07-11

**Authors:** Rita Khusainova, Ildar Minniakhmetov, Olga Vasyukova, Bulat Yalaev, Ramil Salakhov, Darya Kopytina, Raisat Guseinova, Ekaterina Dobreva, Galina Melnichenko, Ivan Dedov, Natalia Mokrysheva

**Affiliations:** Genomic Medicine Laboratory, Endocrinology Research Centre, Moscow, Russia

**Keywords:** childhood obesity, epigenetics, gene–environment interactions, genetic factors, monogenic obesity, polygenic obesity, syndromic obesity

## Abstract

Obesity is a chronic metabolic disease characterized by excessive accumulation or uneven distribution of fat in the body, which poses a serious threat to health. Obesity significantly increases the risk of developing сonditions such as type 2 diabetes, coronary heart disease, hypertension, obstructive sleep apnea, and some types of cancer. The prevalence of obesity, especially in childhood, has increased significantly worldwide over the past few decades. The World Health Organization predicts that 250 million children and adolescents aged 5–19 years will be obese by 2030, which indicates a global problem with far-reaching consequences. Advances in genomic technologies have led to the identification of multiple genetic loci associated with the disease ranging from severe cases with early onset to common multifactorial polygenic forms. Epigenetic changes driven by dietary and lifestyle factors are now recognized as crucial contributors to obesity. These modifications can alter gene expression and thereby link environmental influences to the observable clinical features of the disease. Significant progress has been made in deciphering the genetic architecture of obesity, particularly in pediatric populations. However, further advancement requires integrative multiomics analyses that encompass genomic, epigenomic, transcriptomic, proteomic, metabolomic, and microbiome data. To better understand the complex molecular underpinnings and clinical variability of obesity, researchers are increasingly applying methods from machine learning and artificial intelligence. These technologies help analyze large-scale genomic and phenotypic datasets, allowing for the identification of biological pathways involved in weight regulation. In the future, this may support the design of individualized diagnostic tools and targeted treatment plans that reflect a patient's genetic profile, lifestyle, and environmental exposures. To implement the principles of personalized and precision medicine in the treatment of obesity, it is crucial to identify risk profiles by assessing multiple contributing factors. This approach not only enables the prediction of an individual's risk of obesity and its associated diseases but also facilitates the optimization of treatment based on the patient's genetic profile. This study provides a comprehensive overview of the current understanding of childhood obesity, including its prevalence, genetic determinants, and pathophysiological mechanisms. It highlights the contribution of genetic factors to hereditary and syndromic forms, the role of gene–environment interactions (including nutrition and environmental pollutants), and the influence of epigenetic modifications on metabolic disturbances associated with polygenic obesity.

## 1. Introduction

Overweight/obesity in childhood leads to various disorders and diseases throughout a person's life including hypertension, hypercholesterolemia, and insulin resistance [1], as well as to numerous nonmetabolic comorbidities such as several types of cancer [2], and is the cause of early mortality [3]. In addition, mechanical problems stipulated by increased body weight can provoke the risk of developing osteoarthritis [4] and sleep apnea [5]. The recent COVID-19 pandemic has shown that people with obesity are at increased risk of severe illness and hospitalization [6], highlighting the impact of excess body weight on the course of infectious diseases, especially viral infections [7].

Overall, obesity, especially in childhood, is a serious global health problem, reducing life expectancy and the quality of life; for this reason, identifying risk factors is critical to developing prevention and treatment programs [8]. In addition, it is recommended to consider socio-demographic differences in dietary quality and weight status in the fight against obesity to reduce severe forms of the disease. According to Zhao and Araki, adults with severe obesity demonstrated lower overall scores on the Healthy Eating Index (HEI-2015). Moreover, non-Hispanic Black individuals had lower dietary quality compared to non-Hispanic Asians; women had better dietary quality than men; older adults had better dietary quality than younger individuals; and adults with higher education (college level or above) had better dietary quality than those with less than a high school education [9]. Furthermore, air pollutants can lead to metabolic disorders in the body by inducing adipose tissue inflammation, altering immune responses, and increasing oxidative stress levels in the human body. A recent review on the role of pollution in the development of obesity provides evidence that, in most in vivo and in vitro studies, exposure to heavy metals, preservatives, and pesticides contributes to weight gain [10]. However, several studies have reported contradictory findings, including weight loss, reduced stress response due to exposure to pollutants, no effect on body weight, and no stress response [11].

There is clear evidence that environmental factors significantly contribute to the risk of obesity, including sedentary behavior, the consumption of calorie-dense and nutrient-poor foods, and reduced energy expenditure. However, genetic predisposition plays a substantial role in the development of obesity and can strongly influence an individual's response to environmental conditions [12]. Heritability estimates for obesity and overweight range from 70% to 80%, based on findings from studies involving families [13], twins [14], and adopted children [15]. Extensive evidence also links parental obesity with increased weight in offspring and heightened risk of cardiometabolic complications in adulthood [16].

While genetic discoveries have provided important insights, they do not fully account for the rapid rise in obesity prevalence. Increasing attention has therefore turned to epigenetic mechanisms, which alter gene expression without changing the underlying DNA sequence. These modifications are thought to mediate the biological impact of environmental exposures, such as diet and lifestyle, on health outcomes [13]. Epigenetic changes—including altered DNA methylation—may represent a key interface between genetic susceptibility and environmental triggers like energy intake, physical inactivity, and tobacco exposure [17].

Modern lifestyle changes have facilitated the emergence of obesogenic environments—settings characterized by physical inactivity and frequent intake of ultraprocessed, energy-dense foods [18]. Such environments are considered major contributors to the global obesity epidemic, although the causal pathways remain difficult to disentangle due to the bidirectional influence of environmental and biological systems, including epigenetic regulation.

In parallel, the gut microbial ecosystem has emerged as another important factor in obesity pathogenesis. The gut microbiota comprises a complex community of bacteria, archaea, viruses, fungi, and protozoa that inhabit the human gastrointestinal tract. In contrast, the term “gut microbiome” refers specifically to the collective genetic and functional potential of these microorganisms [19]. This ecosystem is now viewed as a metabolically active interface that produces a variety of biologically active molecules—including enzymes, hormones, and metabolites—that influence host physiology. Alterations in microbiota composition have been associated with obesity and related metabolic conditions in numerous studies [20]. Recent evidence has also revealed distinct microbial profiles in children with normal weight, overweight, and obesity, along with associations between exposure to specific xenobiotics and changes in microbial composition, body weight, and health outcomes [21].

Genetic predisposition, in combination with environmental factors contributing to obesity, introduces individual variability in body weight regulation, emphasizing the complex and dynamic etiology underlying the pathophysiology of obesity. From a genetic perspective, obesity can be classified into two main forms: syndromic obesity, which is generally associated with chromosomal abnormalities (e.g., Prader–Willi syndrome [PWS], Fragile X syndrome, Alström syndrome, Cohen syndrome, Down syndrome, and pseudohypoparathyroidism, among others), and nonsyndromic obesity, which includes both monogenic and polygenic forms [22]. The monogenic form of obesity is caused by pathogenic variants of certain genes and is usually inherited in a Mendelian manner with early severe heterogeneous manifestation of phenotype. By contrast, common obesity is polygenic—it results from the combined influence of many genetic variants (potentially hundreds), each of which individually has only a small effect on a person's predisposition to gain weight. Currently, numerous genetic loci associated with both syndromic and monogenic severe forms of obesity, as well as the common polygenic form, have been identified. In addition, epigenetic analyses of genome modifications, which do not involve changes in the underlying DNA sequence, have revealed key mechanisms in the development of obesity.

Despite significant advances in obesity genetics research, the full genetic architecture of obesity has not yet been determined. Meta-analyses of genome-wide association studies (GWAS) have shown that the variants identified to date explain less than 6% of the observed variability in body mass index (BMI) [23], indicating the existence of “missing heritability” that has yet to be identified. As a consequence, the search for DNA markers is actively ongoing.

Environmental factors such as diet and exercise can alter epigenetic modifications and, consequently, affect gene expression. Clinical variables associated with obesity have been significantly linked to epigenetic changes in various cell types, including skeletal muscle, liver, and adipose tissue [24]. Moreover, such epigenetic modifications are reversible, making them promising targets for the development of targeted therapies.

However, key questions regarding the molecular architecture of severe childhood obesity remain unresolved. There are population- and region-specific variations not only in the spectrum of mutations in known causative genes but also in the genetic diversity contributing to monogenic forms of severe obesity. Polygenic forms of obesity also require further comprehensive investigation of genetic, epigenetic, and environmental factors, taking into account the genetic background of indigenous populations across different regions of the world. Such research will facilitate the optimization of diagnostic approaches and contribute to the development of new therapeutic products targeting novel genetic loci and associated biochemical pathways.

The objective of this study is to provide a comprehensive overview of childhood obesity prevalence, evaluate the genetic contribution to hereditary and syndromic forms, investigate gene-environment interactions, and examine epigenetic modifications contributing to metabolic disorders in polygenic obesity.

## 2. Prevalence of Obesity in Different Countries of the World

According to the World Health Organization (WHO), the prevalence of obesity almost tripled in the period between 1975 and 2016 worldwide and is expected to double again in the next 25 years [25]. The researchers forecasted that, should post-2000 obesity trends persist, global obesity prevalence would rise to 18% in men and exceed 21% in women by 2025. Additionally, severe obesity in women would outstrip underweight prevalence, indicating an extremely low likelihood of meeting the global goal of stopping the rise in obesity by 2025 and reverting prevalence to 2010 levels [26]. By 2020, nearly 39 million children under five years of age were overweight or obese worldwide [27]. The WHO predicts that the number of young children who are overweight or obese will reach 70 million worldwide by 2025, along with the fact that 250 million children/adolescents aged 5–19 years will be obese by 2030. These facts highlight a global problem with far-reaching consequences.

However, there are regional and ethnic differences in obesity rates. In particular, Southeast Asian countries have one of the lowest rates of overweight and obesity in the world, ranging from 2.2% to 15.5%. Meanwhile, in some large South Asian countries including Afghanistan, Bangladesh, India, and Sri Lanka, the prevalence of overweight or obesity among adults ranges from 22.4% to 52.4% [28]; in Arab countries, obesity varies from 4% to 55% [29].

In Korea, the overall prevalence of obesity in 2021 was 38.4%, more specifically 49.2% in men and 27.8% in women, that is, 1.27 times higher than it was in 2012. In particular, among children and adolescents, rates rose from 9.7% in 2012 to 19.3% in 2021, with a greater increase among boys [30]. In the United States in the period of 2017–2020, the prevalence of obesity reached 41.9% among adults over 20 years of age, while 19.7% accrued to the approximately 14.7 million children and adolescents aged 2–19 years [31]. In China, the overweight and obesity rates among children and adolescents aged 6–17 years reach 19.0% (11.1% for overweight and 7.9% for obesity) [32]. In the Middle East, the prevalence of childhood obesity is relatively high compared to other regions of the world, amounting to 15%–25% [33]. Greece ranks first in the European Union for childhood obesity [34].

Currently, an increasing body of evidence suggests ethnic differences in genetic predisposition to obesity; however, many genetic variants responsible for these differences have yet to be identified. These differences may be influenced by variations in lifestyle, socioeconomic status, access to healthcare, social marginalization, or discrimination. Alternatively, they may reflect ethnic differences in genetic susceptibility to obesity. A study on immigrant health in Oslo identified the highest prevalence of obesity among Turks (51%) and the lowest among Vietnamese (2.7%). Notably, BMI differences persisted even after adjusting for sociodemographic and environmental factors, despite all groups residing in the same country [35]. Investigating genetic diversity among ethnic groups is crucial for reconstructing the evolutionary mechanisms that have shaped genetic predisposition or protection against obesity in modern human populations. In recent years, several GWAS on obesity-related traits have been conducted in European, East Asian, and African populations, identifying both European obesity loci and novel, ethnically specific loci.

Populations in low- and middle-income countries are undergoing a rapid transition from traditional to Western dietary and lifestyle patterns, particularly in urban settings. A stronger association has been found between the *FTO* gene, body weight, and urban residency compared to rural living [36], suggesting strong gene-environment interactions and the manifestation of genetic susceptibility under changing environmental conditions. At present, the rising prevalence of obesity worldwide likely reflects a shift away from traditional dietary patterns in many indigenous populations across various regions.

The prevalence of obesity in Russia, according to the first national epidemiological cross-sectional study (NATION), was 31% and proved to be significantly higher in women in comparison to men among participants aged 45 years and older [37]. One of the largest population-based studies in the Russian Federation, conducted in 2004 and involving 13,700 children aged 6–18 years (the average age of the individuals was 13 years) from 6 regions, including Tver, Rostov, Tula, Bryansk, Kaluga, and Oryol regions and Sakhalin Island, revealed overweight in 5.5%–11.8% of cases, while the obesity was found in 5.5% of children from rural areas and in 8.5% of children from urban areas [38]. A study for the period of 2017–2018 conducted in Moscow as part of the Childhood Obesity Surveillance Initiative (COSI) program, which included 2166 seven-year-old children, found that 27% of boys and 22% of girls were overweight, and 10% and 6% among the respective child groups were obese [39]. According to other research, in the general population of children in Russia, aged 2–18 years, the prevalence of overweight amounts to 18% and obesity to 9.1%; more specifically, in boys, these rates are 20.4% and 10.4%, respectively, while in girls, these rates are 15.4% and 7.6% [40].

Thus, the extent of the increase in the incidence of obesity in the entire population and especially in children poses a serious threat to the health of future generations worldwide, and this problem requires close attention from specialists in various fields, including geneticists, to develop effective algorithms for the prevention and treatment of the disease based on the genetic profile of patients.

## 3. The Problem of Assessing the Anthropometric Parameters of Obesity

The search for genetic factors of obesity takes into consideration the anthropometric criteria of assessing excess body weight, which is typically determined by the BMI and calculated by the following formula: weight (kg) divided by height (m^2^). Determining obesity in children and adolescents based on the BMI has been shown to be more challenging than in adults, whereas there is no single threshold value that can be applied to different age groups of children [41]. Adjustment of rates for age and gender is important as BMI value ranges widely during childhood and adolescence, particularly during puberty [42]. Concurrently, to determine childhood obesity, height standards are used, which may also be indicated by BMI z-scores and body conditioning scores [43].

The BMI does not distinguish between lean and fat mass and does not take into account the distribution of body fat, which can lead to misinterpretations of individual health risks. To identify obesity and its association with potential comorbidities, other indices such as waist and neck circumference, waist-to-hip ratio, and waist-to-height ratio are used as better independent indicators of central obesity and predictors of cardiometabolic diseases [44]; besides, they are more precisely associated with overall mortality [45].

Advances in technology have enabled the classification of human body composition based on body fat using more accurate measurement methods, including dual-energy x-ray absorptiometry (DXA), air displacement plethysmography (BodPod), bioimpedance analysis, computed tomography (CT), magnetic resonance imaging (MRI), and ultrasound. Among these methods, bioelectrical impedance analysis (BIA) is the most practical and accessible technology for routine body composition assessment across various fields, including body fat measurement. The cost and time associated with measuring body composition using BIA are significantly lower than those of methods such as DXA or hydrodensitometry, which was previously the standard method. Under controlled conditions involving healthy men and women, a commercially available multifrequency BIA system (InBody 770, Cerritos, California, USA) has demonstrated high precision and reliability in measuring key components of body composition, including body fat percentage [46].

The development of bioinformatics resources enabled the assessment of the cause-and-effect relationships between anthropometric indicators and clinical laboratory parameters of obesity. The investigations based on univariate and multivariate Mendelian randomization and controlling on gender, age, and multiple life-course exposures to obesity revealed a null association of obesity with gestational variables, a negative association with birth weight, and a positive association with childhood BMI. In female participants, higher birth weight causally reduced the fasting insulin level, while in male participants, it reduced the fasting glucose level. Analysis in time revealed the absence of the independent effects of birth weight in female participants and their more pronounced exhibition in male participants. The independent effects of childhood BMI were attenuated in both genders while the independent effects of adult traits differed by gender [47].

Thus, the assessment of the anthropometric parameters of obesity in humans, especially in childhood, is a complex task; as a consequence, further optimization of reference standards is required for predicting health risks associated with obesity. Considering the genetic heterogeneity of obesity, standardization for the assessment of anthropometric parameters of the disease is a prerequisite for the successful identification of genetic factors of the disease.

## 4. Genetic Determinants of Obesity

Twin studies have estimated the heritability of obesity within the range from 40% to 75% [48]. Many of the candidate genes and metabolic pathways associated with body weight regulation were initially identified in obese mice, which spontaneously develop severe hyperphagia and obesity [49]. In particular, leptin deficiency was associated with the development of severe obesity in mice, and the genes encoding leptin and the leptin receptor (LEPR) were further identified and cloned [50]. Friedman et al. identified leptin as an adipocyte-derived hormone whose deficiency leads to severe obesity in ob/ob mice and proposed its name [51]. The importance of leptin has also been demonstrated by studies on mutations in the genes encoding leptin (ob) and its receptor (db) in mice, which reduce leptin sensitivity and may contribute to leptin resistance in humans [52]. Leptin interacts with its receptor to regulate various physiological functions in hypothalamic neurons and different tissues, activating the expression of multiple genes, including pro-opiomelanocortin (POMC). POMC undergoes proteolytic cleavage, releasing α-, β-, and γ-melanocyte-stimulating hormones (MSH), which serve as mediators of the leptin hormonal signal. POMC undergoes proteolytic cleavage, releasing α-, β-, and γ-MSH, which serve as mediators of the leptin hormonal signal. MSH interacts with MC3R and MC4R on the postsynaptic membrane of neurons, leading to appetite suppression in both animals and humans, stimulating fat utilization in energy metabolism, and preventing excessive fat accumulation [53]. Loss-of-function mutations in genes such as *LEP* (which encodes leptin), *LEPR* (LEPR), *POMC*, or *MC4R* disrupt the normal production or function of those proteins. As a result, affected individuals develop an excessive appetite that ultimately leads to obesity. To date, several mutations in the *LEP* gene and a series of mutations in the *LEPR* gene have been identified in humans. These mutations cause severe obesity in homozygotes but have minimal or no impact on physiological function in heterozygotes. In addition, the melanocortin pathway, which regulates body weight, was first identified in mice; subsequently, numerous candidate genes implicated in obesity have been identified. All of these genes were identified as candidate genes for studying the molecular pathogenesis of obesity in humans. Specifically, researchers identified individuals with congenital leptin deficiency [54], mutations in the gene encoding the LEPR [55], and mutations in multiple genes encoding components of the melanocortin pathway, including *PCSK1* [56], *MC4R* [57], and *POMC* [58]; all these genetic alterations led to early-onset severe obesity.

However, the most common form of obesity is polygenic or multifactorial one, which is stipulated by hundreds, perhaps thousands, loci of independent single-nucleotide polymorphism (SNP) distributed throughout the human genome. Therefore, this form has a complex pattern of inheritance, typical for common phenotypes. Since the first publication of the results of GWAS in 2007, many new pathogenetic links in the development of obesity and overweight have been identified. Moreover, the expression of genes that control the pathogenesis of monogenic obesity may be partially affected by the predilection to polygenic obesity in a particular individual [59].

## 5. Genetic Architecture of Severe Childhood Obesity

**Figure 1:**
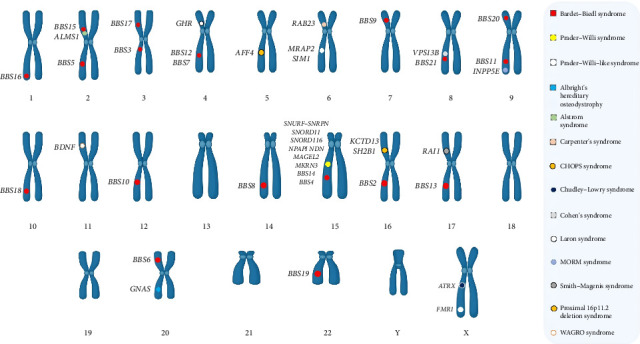
Chromosomal map of common syndromic forms of obesity. (The drawing is an original creation by the author and was made using BioRender software).

Severe obesity in childhood can present as part of rare hereditary syndromes, which are characterized by a combination of marked weight gain, specific developmental, cognitive, and physical abnormalities, and, often, multiple organ involvement (Figure 1). While the clinical spectrum of these disorders is broad, common features include early-onset obesity, dysmorphic traits, and varying degrees of intellectual disability. Recognizing these syndromic forms is crucial, as their management and prognosis differ fundamentally from those of more common, multifactorial obesity [60–63].

Syndromic obesity encompasses a diverse group of disorders arising from disruptions in a variety of genes involved in neurodevelopment, hormonal regulation, and metabolic pathways. The classical examples include PWS and Bardet–Biedl syndrome, but many additional, often extremely rare, conditions have been described. Recent research continues to uncover new genetic loci and pathogenic mechanisms underlying these syndromes, leading to a growing appreciation of both their clinical and genetic heterogeneity [64–66]. This diversity frequently leads to diagnostic ambiguity, as patients may present with overlapping clinical features but possess distinct molecular etiologies.

A notable challenge in clinical practice is the differentiation of syndromic obesity from more prevalent monogenic and polygenic forms. While monogenic nonsyndromic obesity (such as mutations in the *MC4R*, *LEP*, or *POMC* genes) generally presents with severe hyperphagia and weight gain in early life but otherwise normal neurocognitive development, syndromic forms typically display additional systemic or neurological features. Accurate distinction is vital, as it informs both genetic counseling and patient management strategies. The spectrum and function of genes involved in monogenic forms of obesity are given in Table 1.

**Figure 2:**
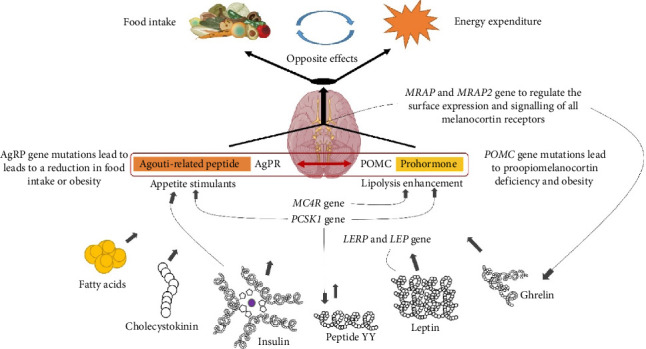
Involvement of various genes in the regulation of the leptin-melanocortin pathway. AgRP and POMC in the arcuate nucleus of the hypothalamus are two upstream elements in the endocrine regulation of the melanocortin pathway. They integrate and distribute central or peripheral information from hormonal and neuronal signals, including fatty acids (FA), cholecystokinin (CCK), peptide YY (PYY), leptin, insulin, etc. (The drawing is an original creation by the author and was made using BioRender and Microsoft Office PowerPoint software).

The same genetic defect can manifest with varying severity or associated features among different patients—even within a single family—due to additional genetic, epigenetic, and environmental modifiers [79] (Figure 2). For instance, distinct phenotypic patterns of syndromic obesity have been documented in various ethnic populations, sometimes leading to underrecognition or misdiagnosis [80]. This variability is further complicated by rare phenomena such as somatic mosaicism, variable penetrance, and differences in X-chromosome inactivation, all of which may obscure the clinical picture [81, 82].

The clinical manifestations of these syndromes may also be influenced by therapeutic interventions. Growth hormone replacement in patients with PWS, for example, can significantly improve growth patterns and body composition, potentially altering the classic presentation and complicating diagnosis [83–87].

Advances in molecular genetics over the last 2 decades have revolutionized the classification and understanding of syndromic obesity. Disorders once grouped together on the basis of shared clinical features have often proven to be genetically distinct, while others—previously considered separate entities—have been unified by the discovery of common molecular defects [88–90]. These developments have important implications for both nosology and individualized care.

Emerging genetic technologies such as whole-exome sequencing, high-resolution chromosomal microarrays, and targeted gene panels have greatly facilitated the identification of causal variants in affected individuals, even when classical features are absent or ambiguous. This approach has also led to the recognition of novel syndromic entities and expanded the phenotypic spectrum associated with known genes [81, 82, 91–99]. As a result, the boundaries between syndromic, monogenic, and even complex forms of obesity are increasingly seen as fluid rather than rigidly defined.

The inheritance of syndromic obesity syndromes is diverse, including autosomal dominant, autosomal recessive, X-linked, and even digenic or oligogenic patterns [91, 92, 98]. In certain conditions, such as Bardet–Biedl syndrome, genetic evidence suggests that pathogenic variants in multiple genes may be required to manifest the full clinical phenotype, although such models remain controversial [93]. The impact of gene modifiers, epigenetic factors, and environmental interactions on expressivity and penetrance is an active area of research, exemplified by conditions like Cornelia de Lange syndrome [99] and various atypical presentations related to mosaicism or skewed X-chromosome inactivation [81, 82].

Large-scale sequencing studies have identified new rare variants implicated in syndromic and monogenic forms of severe childhood obesity. Examples include pathogenic mutations in *P4HTM*, which have been associated with complex phenotypes involving hypotonia, neurodevelopmental delay, and fatal complications in consanguineous families [75, 100]. Similarly, rare variants in genes such as *KSR2* and *MRAP2* have been shown to disrupt key metabolic signaling pathways, resulting in profound effects on energy homeostasis, appetite regulation, and insulin sensitivity [71, 101, 102]. The genetic landscape continues to expand as new loci and candidate genes are identified and functionally characterized.

Other recently described cases involve pathogenic alterations in the *PHIP*, *BDNF*, and *NTRK2* genes, which highlight the overlap between neurodevelopmental disorders and severe obesity [103–109]. The presence of developmental delay, intellectual disability, or additional neurological features alongside early-onset obesity should prompt consideration of these rare genetic causes.

Given the broad genetic and phenotypic heterogeneity of syndromic obesity, a systematic approach to evaluation is essential. This includes comprehensive clinical assessment, detailed family history, and the judicious use of advanced genetic testing platforms. Early and precise identification of the underlying etiology has direct implications for personalized management, family counseling, and the development of novel therapeutic strategies. As research advances, it is likely that the current boundaries between syndromic, monogenic, and complex obesity will continue to blur, necessitating ongoing revision of classification systems and clinical guidelines.

## 6. Genetic Markers of Multifactorial Forms of Obesity

Obesity in children and adults is predominantly a polygenic condition influenced by multiple genetic and environmental factors. Genetic studies aiming to identify obesity predisposition began in the late 1990s, with GWAS significantly advancing our understanding. Early GWAS efforts in 2007 identified variants in the *FTO* gene as having the most substantial effect on BMI. Subsequently, over 1000 genetic loci associated with obesity have been discovered; nevertheless, these variants collectively explain only about 5% of BMI variability, highlighting the challenge known as “missing heritability” in obesity genetics [23, 110].

A major contribution to obesity genetics was made by the GIANT consortium, initially analyzing data from over 330,000 individuals and identifying almost one hundred SNPs strongly associated with BMI. By integrating additional data from the UK Biobank, the study sample expanded to more than 800,000 participants, revealing numerous additional loci. Such extensive analyses have substantially enhanced insights into the influence of common genetic variants on adult obesity [23, 111]. Concurrently, GWAS analysis on childhood obesity has also progressed after eliminating several challenges, including difficulties in recruiting cohorts, signing informed consent and ethical guidelines, as well as difficulties in measuring physical parameters, collecting blood samples from children, and defining and measuring childhood obesity parameters [112].

Early genetic research in pediatric obesity primarily explored whether obesity-associated variants identified in adults also impacted weight in children. Notably, polymorphisms such as rs9939609 within the FTO gene and rs7566605 within INSIG2 were studied extensively in childhood cohorts. Variants in the FTO gene demonstrated particularly consistent associations across pediatric studies, underscoring its prominent and stable role in weight regulation during early development [113, 114]. The results of 13 BMI-associated loci previously reported in adults were evaluated in a pediatric cohort of 6078 European children with measured BMI [115]; and identical studies were conducted among children of African American, South African, and South Asian origins [116–118]. These studies have shown that some loci associated with obesity in adults were associated with BMI in children, but there were limitations.

A meta-analysis combining data from 23 pediatric GWAS, involving 8348 obese children and 12,401 controls, identified nine SNPs significantly associated with obesity in youths aged 2–18 years. Among these, two novel SNPs reached genome-wide significance: one located near the *OLFM4/PCDH8P1* genes (rs9568856, *p*=1.82 × 10^−9^) and another within the *HOXB5* gene (rs9299, *p*=3.54 × 10^−9^10) [68]. In April 2013, GWAS were conducted among 1509 children with severe obesity and 5380 controls; evaluating 29 SNPs (*p* < 1 × 10^−5^) in another 971 children with severe obesity and 1990 controls revealed nine SNPs associated with severe obesity, and five SNPs in the genes *RPL31P12, PRKCH, LEPR, PACS1,* and *NEDD1* were novel [119].

The largest GWAS on childhood obesity were published in July 2019 [120]. rs2540031 (*p*=0.015) in the *ADCY9* gene, rs6567160 (*p*=8.66 × 10^−5^) in the *MC4R* gene, and rs56094641 (*p*=6.55 × 10^−6^) in the *FTO* gene have most significant risk effect in Asian, African, and Hispanics populations [121].

Meanwhile, a number of GWAS have contributed to the comprehension of the effect of age and DNA loci on the formation of BMI or obesity in children according to childhood growth trajectories.

It is important to notice that active investigations are now underway to functionally annotate the associations found. Functional characterization of GWAS-identified obesity loci has elucidated diverse biological mechanisms through which genetic variants influence weight regulation. For instance, the well-studied rs1421095 variant near the *ARID5B* gene in the FTO locus modulates the expression levels of *IRX3* and *IRX5*, both of which are critical regulators of adipocyte differentiation and energy expenditure processes [122]. Another prominent locus, *TMEM18*, encodes a transmembrane protein with poorly understood function. Knockout models in *Drosophila* have shown its involvement in insulin and glucagon signaling [123], while experiments in mice demonstrated that its deletion leads to hyperphagia and weight gain [124]. Variants in *CADM1* and *CADM2*, which encode synaptic adhesion molecules, appear to influence hypothalamic circuits involved in energy balance and insulin sensitivity [125]. Additionally, regulatory sequences upstream of the *NEGR1* gene affect binding of the transcriptional repressor *NKX6.1*, and disruptions in this region are associated with increased *NEGR1* expression and reduced muscle mass in animal models [126], although conflicting results have also been reported [127].

To clarify how specific genetic variants contribute to obesity risk, researchers have applied methods such as stratified linkage disequilibrium regression and cell-type-specific heritability estimation. These analyses helped prioritize 19 genetic signals from childhood obesity GWAS, leading to the identification of potential effector genes—particularly within the *BDNF, ADCY3, TMEM18*, and *FTO* regions—based on gene activity in tissues like muscle and pancreatic beta cells. In the TMEM18 locus, the gene *ALKAL2* emerged as a candidate regulator of inflammation and neuronal signaling. These findings were reinforced through colocalization with expression quantitative trait loci (eQTL) data from the genotype-tissue expression (GTEx) project, indicating the involvement of immune and neurological pathways in pediatric obesity [128].

Although GWAS has revolutionized the discovery of genetic markers associated with obesity, it possesses certain inherent limitations. Notably, GWAS typically fails to identify rare variants or variants with smaller effect sizes, and consequently, it has relatively limited individual predictive capacity. Moreover, different GWAS studies often yield contradictory results, and heritability estimates may be inflated due to environmental influences or genetic interactions. Population stratification and admixture should also be accounted for, as polymorphism frequencies may vary depending on ancestral origin. To address this gap, a novel approach known as genetic risk scoring (GRS) or the polygenic risk score (PRS) has been developed. This method enables the aggregation of multiple risk loci into a single predictive model [129]. Furthermore, GWAS and meta-analyses have not fully accounted for BMI variance. Omics technologies may help elucidate the mechanisms of obesity by leveraging advancements in genomics, epigenomics, transcriptomics, proteomics, metabolomics, and microbiomics. Current efforts focus on integrating various omics biomarkers into multiomics approaches to refine phenotype characterization and implement targeted precision prevention. However, further genomic studies are required to develop bioinformatics platforms capable of generating, analyzing, and interpreting multiomics data as a foundation for designing precise obesity prevention strategies.

Comprehensive functional analyses of GWAS-derived obesity loci promise valuable insights into the underlying genetic etiology. However, many obesity-associated genetic loci remain incompletely characterized, with precise molecular mechanisms still needing elucidation. Identifying the causal variant(s) at each of these loci and then connecting them to the causal effector gene(s) remains challenging. To achieve this goal, various methods are used, including SNP enrichment analysis, molecular signature profiling, colocalization analysis, transcriptome-wide association studies (TWAS), regulome-wide association studies (RWAS), integration of PRS with functional annotations, and using different types of quantitative traits (eQTL, sQTL, and 3'aQTL) and tissue types. More recently, machine learning and artificial intelligence (AI) networks have also been used for effector gene prediction. These findings require further functional evaluation, including the involvement of CRISPR-based approaches. Furthermore, using gene ontology to analyze gene overlap in comorbidities such as diabetes and hypertension may provide new insights into key pathways involved in the pathogenesis of obesity.

## 7. Epigenetic Aspects of Obesity

Recent research has significantly advanced our understanding of epigenetic markers associated with obesity. Epigenetic modifications, distinct from genetic variations, offer valuable insights into individual susceptibility to obesity, as these alterations can be inherited through germline transmission and influenced by environmental exposures [130].

Investigations involving newborn cohorts in the Netherlands demonstrated a connection between parental nutrition prior to conception and inheritable epigenetic alterations in offspring [131]. Additionally, obesity has been linked to distinct epigenetic changes, including DNA methylation, histone modifications, and alterations in noncoding RNA (ncRNA) profiles within spermatozoa and oocytes [132]. The negative impact of obesity on oocytes was studied mainly in model obesity organisms, where changes in histone modifications and DNA methylation were revealed under these conditions [132].

Dietary patterns such as high-fat or low-protein intake, as well as interventions like bariatric surgery, have been shown to induce changes in RNA expression profiles and DNA methylation in sperm cells, potentially contributing to metabolic disturbances, including insulin resistance and altered weight trajectories in offspring [133]. Longitudinal and prospective studies on bariatric surgery in adolescents have established that this procedure is both safe and effective for treating obesity in children. Moreover, the complication rate of bariatric surgery in adolescents is lower than that in adults [41].

DNA methylation, particularly at cytosine residues within CpG dinucleotides, is among the most common epigenetic modifications. Typically, methylation at promoter or regulatory regions is associated with transcriptional repression [134], whereas methylation within gene bodies frequently correlates with active gene transcription [135].

Studies have demonstrated that elevated methylation levels of the *LEPR* gene correlate with insufficient maternal weight gain during pregnancy, subsequently influencing protein expression patterns in neonatal umbilical vein samples [136]. Human and animal studies have shown that high-fat and low-protein diets during pregnancy led to changes in the epigenetics of specific genes and resulted in obesity and related comorbidities in offspring, which persisted into adulthood. Conversely, healthy diets have beneficial epigenetic effects.

Genome-wide DNA methylation analysis was performed in peripheral blood samples from 41 children with typical obesity and 31 normal-weight children from the control group to identify differentially methylated sites (DMSs). The overall DNA methylation level in the obese group was significantly lower than that in the norm. A total of 241 DMSs were identified, and functional pathway analysis showed that differentially methylated genes were primarily involved in lipid, carbohydrate, and amino acid metabolism, as well as in disease development and other biochemical and physiological pathways. The characteristic DMSs in the genes of mitochondrial transcription factor A (*TFAM*) and piezotype mechanosensitive ion channel component 1 (*PIEZO1*) were identified as CpG-cg05831083 and CpG-cg14926485, respectively. In addition, the methylation level of CpG-cg05831083 was significantly correlated with the BMI and vitamin D level [137]. The authors conclude that methylation of CpG-cg05831083 and CpG-cg14926485 sites may potentially serve as biomarkers of typical obesity in children.

ncRNAs have emerged as important contributors to obesity pathogenesis and are considered promising candidates for early diagnostic biomarkers. The pathogenesis of metabolic diseases is associated with the expression of various microRNAs that are small ncRNAs involved in post-transcriptional gene regulation. In particular, 221 out of 1736 loci associated with obesity overlap with microRNAs, 54 of which are located in human housekeeping genes, while others are found in large intergenic regions and introns [138]. Among them, the most notable are miR-335 (localized within the *MEST* gene, a homolog of the mesoderm-specific transcript), miR-378 (located within the peroxisome proliferator-activated receptor gamma coactivator 1 beta gene Ppargc1b), and miR-33 (found within the sterol regulatory element-binding transcription factor 2 gene Srebf2), among others. Additionally, microRNAs let-7a, let-7b, let-7c, let-7e, let-7f, miR-103, miR-10b, miR-125a, miR-125b, miR-143, miR-23a, miR-23b, miR-26a, and miR-99b have been shown to influence fat deposition in humans. MicroRNAs have been reported to be able to modulate pathways, which control adipogenesis; the latter is disturbed in obesity [139]. In this regard, dysregulation of microRNAs may be involved in metabolic processes that contribute to obesity [140]. Concurrently, microRNAs prevent the translation of mRNA into proteins leading to mRNA degradation or translational repression with subsequent impairments of the regulation of various cellular processes and development of metabolic disorders and obesity [141].

Long ncRNAs (lncRNAs), which are substantially longer than typical ncRNAs, significantly contribute to gene regulatory mechanisms, suggesting their involvement in obesity development and related metabolic complications. It has been shown that various lncRNAs participate in the early and late phases of adipogenesis and are included in the development of obesity-associated complications. Nevertheless, further studies are needed to elucidate the specific functions of lncRNAs in this process, as there are conflicting data. In particular, this concerns maternally expressed gene 3 (Meg3), a novel lncRNA that is capable of both inhibiting and stimulating adipogenesis [142].

Histone modifications constitute another major epigenetic mechanism implicated in obesity development [143]. Among these, histone acetylation and methylation are most frequently studied due to their significant roles in regulating gene expression patterns related to obesity. Histone acetylation results in increased gene expression because DNA becomes more accessible for transcription. In contrast, histone methylation can both enhance and repress gene expression by disrupting or enhancing DNA-histone interactions. In liver cells isolated from high-fat-diet-fed animals, increased histone acetylation was found in the histone 3 lysine 9 (H3K9) and histone 3 lysine 18 (H3K18) sites, leading to increased tumor necrosis factor (TNF—multifunctional proinflammatory cytokine) expression [144]. Investigations have shown that increased TNF expression may be associated with obesity due to its tendency to act as a mediator of insulin resistance [145].

Adult-onset obesity can be significantly influenced by dietary habits and various environmental exposures, with a robust link established between obesity phenotypes and distinct epigenetic signatures. These patterns can be established early in life and shape susceptibility to obesity and its associated diseases in adulthood. Because epigenetic changes can be influenced by environmental factors, they are increasingly recognized as adjustable elements in the development of obesity. Diet, physical activity, and other exposures during sensitive periods of life—such as fetal development or early childhood—may reshape epigenetic patterns and affect metabolic programming. Although many epigenetic marks are dynamic and reversible, some may become fixed and influence disease risk over time. This makes them promising candidates for early biomarkers, particularly in strategies aimed at tailoring nutrition and lifestyle interventions to individual risk profiles [146]. It is well known that diet and increased physical activity are the most effective treatments for obesity, and their influence on epigenetic modifications has been suggested. Due to the novelty of epigenetics, major epigenetic approaches to obesity treatment are primarily conducted in animal models, although human studies also exist. Physical exercise has been shown to induce changes in DNA methylation and mRNA expression in adipose tissue across 39 genes involved in the pathogenesis of obesity and type 2 diabetes [147]. Other researchers have investigated the relationship between global DNA methylation, genetic variants associated with energy balance and lipid metabolism, and weight loss following nonsurgical weight reduction interventions. Their study demonstrated an inverse association between global DNA methylation and weight loss: as weight loss increased, global DNA methylation decreased [148].

Conversely, obesity accelerates hepatic epigenetic aging, known as DNAm age, which is based on the DNA methylation measurement of 353 CpG sites. This effect is irreversible in obese patients following a rapid weight loss intervention and may contribute to liver comorbidities associated with obesity [149]. Thus, epigenetic markers may serve as valuable tools to personalize dietary interventions for weight reduction, enhance patient responsiveness to weight loss interventions, and facilitate the early identification of individuals at higher risk of obesity and metabolic disorders.

**Figure 3:**
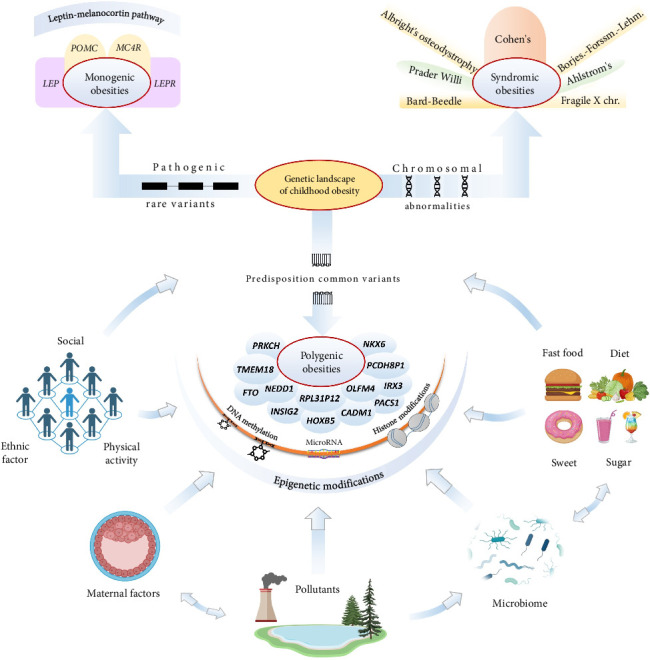
Key components of the genetic architecture of childhood obesity (the drawing is an original creation by the author and was made using BioRender and Microsoft Office PowerPoint software).

Although extensive research has examined epigenetic influences on obesity, further investigation is required to clarify the impact of factors such as hypoxia, chronic inflammation, oxidative stress, and hormonal dysregulation on epigenetic mechanisms governing post-transcriptional gene expression. In addition, large-scale studies should be conducted to determine the relationship between environmental factors such as diet, physical activity, sleep, alcohol consumption, and epigenetic alterations (Figure 3). The modifiable nature of epigenetics makes it a promising route for the prevention and treatment of obesity.

## 8. Systemic Integrative Multiomics Approaches in Obesity Research

With the increasing accumulation of data on the role of genetic, epigenetic, and environmental factors in obesity in general and in children in particular, integrative approaches can combine these research findings to develop effective strategies for obesity prevention and treatment. Currently, studies integrating various biomarkers using multiomics approaches are already being conducted for the comprehensive assessment of risk factors in multifactorial forms of obesity. There is strong evidence to suggest that combining multiomics data with whole-genome and epigenome profiling, using bioinformatics tools—particularly PRS for individuals and Mendelian randomization—will facilitate the development of advanced strategies for the prevention and treatment of childhood obesity. Multiomics profiling of childhood obesity based on methylome, microRNAome, transcriptome, proteome, and metabolome analysis has revealed the complex molecular landscape of pediatric obesity and its associated metabolic dysfunction in European children [150]. Notably, microRNAs, gene transcription clusters, and proteins contributed the most to defining the high-risk cluster. This risk cluster was also associated with a distinct proteomic profile characterized by elevated circulating levels of HGF, IL-6, TNF-alpha, BAFF, IL1-beta, CRP, IL-8, IL-1RA, and MCP1. Furthermore, most pathways related to this cluster were associated with immune system function and inflammation (e.g., immune cell differentiation and activation, Toll-like receptor signaling, MAPK signaling, TNF signaling, JAK/STAT signaling), endothelial function (specifically pathways regulating vascular endothelial growth factor production, nitric oxide biosynthesis, and HIF-1 signaling), and other biological processes. The findings from this largest multiomics study to date identified subgroups of children with distinct phenotypic characteristics based on the variability of factors within this cluster. Among several prenatal factors, pre-pregnancy BMI and maternal levels of perfluorooctanoic acid were the strongest predictors of the high-risk cluster in the North/West European cohort. Undoubtedly, pathogenic variants in key genes involved in lipid and carbohydrate metabolism, neuroendocrine regulation, the pro-opiomelanocortin pathway, as well as several rare chromosomal mutations, in combination with epigenetic modifications, contribute to a complex spectrum of obesity phenotypes with diverse clinical manifestations [151].

A recent cross-tissue multiomics analysis identified inflammatory genes—such as *IL4R, ZNF438*, and *LILRA5*—associated with severe obesity in Latin American populations. These findings underscore the involvement of metabolic, inflammatory, and insulin-related pathways and highlight novel therapeutic targets for obesity treatment [152]. Another study introduced an advanced multiomics strategy to elucidate antiadipogenic mechanisms in adipocytes in the context of probiotic combination therapy. This approach demonstrated effectiveness in identifying novel regulatory pathways and target molecules, reinforcing the potential of multiomics for uncovering probiotic-mediated antiobesity mechanisms [153].

In addition, a study published in 2022 utilized machine learning algorithms to predict obesity based on genome-wide and epigenome-wide gene–diet interactions. The authors identified 21 SNPs, 230 CpG DNA methylation sites in the genes *CPT1A*, *ABCG1, SLC7A11, RNF145*, and *SREBF1*, as well as 26 nutritional factors as key predictors of obesity. These nutritional factors included processed meat, diet soda, French fries, high-fat dairy products, artificial sweeteners, alcohol consumption, and specific nutrients and food components such as calcium and flavonols [154].

A recent study applied AI and machine learning techniques to perform an integrative unsupervised clustering analysis based on proteomic and metabolomic data, aiming to identify subpopulations among individuals with obesity. This approach revealed two distinct clusters: Cluster 1 was characterized by significantly higher mean values of BMI, fasting blood glucose, and inflammatory markers, while Cluster 2 showed elevated levels of total cholesterol and high-density lipoproteins. Pathways associated with cell growth, lipogenesis, and energy expenditure were enriched in Cluster 1, whereas Cluster 2 was primarily linked to inflammatory response and insulin resistance pathways. The authors suggested that these distinct clusters may reflect differences in dietary or behavioral factors, or represent varying stages of metabolic deterioration [155].

Thus, these studies have highlighted the potential of a systems approach to analyzing genomic research findings in obesity. However, this field is in its early stages, and multiomics data remain scarce due to their high cost, the lack of technical infrastructure, and a shortage of competent specialists in bioinformatics and molecular genetics in many countries. These limitations impede advancements in the comprehensive study of human diseases, including obesity.

## 9. Recent Advances in Obesity Treatment

The study of the pathogenic mechanisms of obesity has opened new opportunities for pharmacological treatment across different obesity types. Currently, GLP-1 analogs have gained widespread use. These drugs were initially developed for the treatment of patients with type 2 diabetes mellitus but have subsequently demonstrated efficacy in weight loss therapy. Among these, liraglutide has the most substantial scientific evidence supporting its effectiveness. Its benefits in treating obesity have been confirmed in randomized controlled trials (RCTs) for managing simple obesity in adults and children over 12 years of age [156]. Another GLP-1 analog, semaglutide, has also shown promising results in treating common obesity in adults and adolescents [157]. According to a double-blind randomized trial conducted in 2022, patients receiving semaglutide experienced significant weight reduction. The average BMI reduction was −16.1% compared to +0.6% in the placebo group. Additionally, substantial improvements in quality of life and lipid profile were observed. Semaglutide was also shown to be more effective in weight reduction compared to other GLP-1 analogs. The efficacy of GLP-1 receptor agonists has also been studied in patients with PWS as part of a 52-week multicenter RCT. Patients received daily liraglutide injections combined with lifestyle modifications, including dietary therapy and physical activity. The study results did not show significant changes in BMI SDS (−0.1 SD); however, a notable reduction in hyperphagia was observed among adolescents receiving treatment [158]. The effectiveness of a daily 3-mg dose of liraglutide was also investigated in patients with monogenic obesity, specifically in 14 carriers of pathogenic variants in the *MC4R* gene compared to 28 patients without mutations. Both groups exhibited equivalent weight loss, approximately 6% of baseline body weight, after 16 weeks of therapy. Currently, no available data exist on the use of GLP-1 analogs in other monogenic forms of obesity [159].

PWS is the most common form of syndromic obesity in children. The development of obesity in this condition is associated with hypothalamic dysfunction, resulting in the absence of satiety, impaired oxytocin signaling, and growth hormone deficiency. Recombinant growth hormone therapy has demonstrated efficacy in patients with this disorder and is recommended from the time of diagnosis, provided there are no contraindications. It has been proven that somatropin therapy improves growth parameters, increases muscle mass, and enhances psychomotor development [160].

A potential therapeutic target for patients with PWS is the ghrelin pathway. Livoletide, a nonacylated ghrelin analog, demonstrated promising effects on eating behavior in an RCT involving 40 PWS patients treated for 14 days [161]. Currently, the effectiveness of ghrelin O-acyltransferase (GOAT), an enzyme that converts ghrelin into its active form, is being evaluated in PWS patients [162].

Research on the leptin–melanocortin pathway by scientists worldwide has led to the development of innovative therapies for monogenic forms of obesity. In 1997, Montague et al. described the first clinical cases of congenital leptin deficiency [67], and by 1999, Farooqi et al. reported the efficacy of recombinant leptin (metreleptin) therapy in patients with this condition [163]. Despite the high efficacy of recombinant leptin therapy in patients with obesity due to congenital leptin deficiency, metreleptin treatment has not been effective in other forms of monogenic obesity.

Since then, scientific efforts to develop treatments for monogenic obesity have led to the creation of melanocortin 4 receptor (MC4R) agonists. Currently, the highly selective MC4R agonist setmelanotide is successfully used in clinical practice, demonstrating efficacy in the treatment of monogenic obesity caused by POMC deficiency, LEPR deficiency, proprotein convertase subtilisin/kexin type 1 (PCSK1) deficiency, as well as Bardet–Biedl syndrome. The therapy has been approved by the FDA for the treatment of children over 2 years old with a genetically confirmed diagnosis of the four aforementioned disorders [164].

Active research is ongoing to develop RNA-based therapies for obesity. For example, combined treatment with pioglitazone and *FGF21* mRNA significantly reduced body weight, fasting glucose levels, insulin levels, and improved lipid metabolism in an animal model [165].

Furthermore, Wang et al. engineered human white preadipocytes using CRISPR–Cas9 to activate the expression of the thermogenin protein, thereby creating human brown-like (HUMBLE) cells. Transplantation of HUMBLE cells into diet-induced obese mouse models improved glucose tolerance and insulin sensitivity, increased energy expenditure, and reduced weight gain without adverse effects. HUMBLE cells activated endogenous brown adipose tissue through erythrocyte-mediated nitric oxide production and delivery [166].

Thus, molecular genetic research has led to significant progress in the treatment of hereditary forms of obesity, and ongoing studies of the disease's molecular pathogenesis provide hope that personalized therapy will become available for other common forms of obesity.

## 10. Conclusion

Сhildhood obesity represents a heterogeneous group of metabolic disorders driven by genetic diversity in the causes of this pathology. Summarizing the above, the genetic landscape of severe obesity in children can be outlined in broad strokes. Approximately 5%–10% of cases are syndromic and monogenic forms. There is still no consensus on how these two forms differ; it is generally believed that structural variations in small or large chromosomal segments lead to syndromic forms of obesity, whereas monogenic forms are caused by pathogenic variants in genes of the leptin–melanocortin pathway. The most frequent mutations occur in the *MC4R* gene, followed by mutations in *LEPR, POMC, PCSK1*, and *LEP*. Both forms of obesity manifest early in life (before the age of 5) and follow a Mendelian inheritance pattern, with either recessive or dominant modes of transmission. Sporadic cases and variants with incomplete penetrance have also been observed. In addition, genomic imprinting, an epigenetic phenomenon, leads to the inactivation of either the paternal or maternal copy of certain genes, for example, through the methylation of specific cytosine residues in DNA within the oocyte or sperm. The discovery of genomic imprinting has provided an explanation for the peculiarities of development and inheritance in PWS. All other forms of obesity are polygenic or multifactorial, developing under the influence of environmental and epigenetic factors. Epigenetic modifications can occur during specific periods of ontogenesis, such as the prenatal stage, yet manifest later in adult life. Genes associated with susceptibility to multifactorial obesity are also found in monogenic forms, with the key difference lying in the effects of nucleotide sequence alterations: structural changes at the protein level lead to inherited forms, whereas quantitative effects contribute to polygenic variants. Furthermore, environmental factors—including shifts in traditional dietary patterns among various global populations due to globalization, increased consumption of snacks, processed foods, and alcohol—also contribute to the rising prevalence of obesity. Recent advances in understanding the molecular mechanisms of monogenic obesity have led to the development of targeted therapies aimed at overcoming genetic defects. Leptin deficiency is treated with the recombinant human leptin analog metreleptin, while individuals with LEPR, PCSK1, or POMC deficiencies can now be treated with the MC4R agonist setmelanotide.

Despite significant progress, a complete understanding of the genetic architecture of obesity in general, and childhood obesity in particular, remains elusive. The comprehensive application of methodological advancements in genomics, epigenomics, transcriptomics, proteomics, metabolomics, and microbiomics, along with the integration of various omic biomarkers into multiomics approaches, as well as the utilization of machine learning and AI methodologies, can contribute to a more complete understanding of the molecular pathogenesis of obesity and its distinct phenotypes. These advancements will aid in developing algorithms for diagnostics, treatment, and targeted precision prevention, taking into account the genetic structure and environmental factors of populations across different regions of the world. To achieve this, further research must be conducted in various regions, with a primary focus on identifying the molecular and genetic causes of hereditary forms of obesity and assessing their prevalence. This should be followed by the identification of the hereditary component in multifactorial forms of obesity. Additionally, further development of bioinformatics tools, optimization of disease classification criteria, and enhancement of diagnostic algorithms will be required. Ultimately, these efforts will lead to increased efficacy in the treatment and prevention of obesity.

## Figures and Tables

**Table 1:** The spectrum and function of genes involved in monogenic forms of obesity.

Gene	Protein	Function	Mechanism and form of obesity	Type of inheritance	References
LEP	Leptin	An anorectic hormone produced by white adipocytes; its level in the blood decreases on an empty stomach and increases during meals; affects appetite through the hypothalamus	Leptin deficiency leads to severe forms of obesity	Autosomal recessive	[55]

LEPR	Leptin receptor	Leptin binding in the hypothalamus transmits signals from adipose tissue to the central nervous system about sufficient energy reserves	Morbid obesity with early onset; deficiency of gonadotropic, somatotropic, and thyroid-stimulating hormones, immune disorders	Autosomal recessive	[67]

POMC	Pro-opiomelanocortin	It is part of the central melanocortin system	Deficiency leads to lack of adrenocorticotropic hormone, α-melanocyte-stimulating hormone (α-melanotropin, α-MSH), and β-endorphins, causing severe obesity, adrenal insufficiency; red hair is typical	Autosomal dominant and autosomal recessive	[58]

MC4R	Melanocortin 4 receptor	It is a factor involved in the regulation of eating behavior (suppressing appetite) and energy balance	Deficiency leads to increased appetite and eating behavior in children, along with the occurrence of additional comorbidities, mainly related to growth process	Autosomal dominant and autosomal recessive	[58, 59]

ADCY3	Adenylate cyclase 3	Catalyzes the synthesis of cyclic AMP from ATP, mediates the action of hormones involved in the energy control including metabolism of lipids and glucose	Loss of function leads to abdominal obesity, insulin resistance, dyslipidemia, type 2 diabetes, and olfactory impairment due to impaired cyclic AMP (cAMP) signaling	Autosomal recessive	[68]

SIM1	Homolog of *Drosophila* single-minded (SIM) transcription factor	Participates in the MC4R pathway and is considered to be involved in the formation of the supraoptic and paraventricular nuclei of the hypothalamus	Prader–Willi-like syndrome, early-onset morbid obesity, developmental retardation, hypotonia, emotional lability, autistic behavior	Autosomal recessive	[69]

MRAP2	Accessory membrane protein 2 of the melanocortin receptor (MRAP2)	Binds to G protein-coupled receptors and regulates signal transduction by stimulating MC4R-induced cAMP production in response to αMSH	Obesity caused by MRAP2 deficiency is usually not morbid, but is accompanied by the development of hyperglycemia and/or arterial hypertension	Autosomal dominant	[62]

SH2B1	Src homology 2 B adaptor protein 1	Participates in regulation of sensitivity to leptin	Severe childhood obesity with signs of developmental retardation	Autosomal dominant	[70]

KSR2	Kinase suppressor of Ras 2	As an intracellular scaffold protein, it is involved in multiple signaling pathways and is an important regulator of intake and expenditure of energy in the human body	Childhood hyperphagia, decreased basal metabolic rate, low heart rate, and severe insulin resistance	Autosomal dominant	[71]

PCSK1	Proprotein convertase-1/3 (PC 1/3)	Neuroendocrine convertase, a member of the subtilisin-like serine endoproteases family, which converts large precursor proteins into mature biologically active products	Severe childhood obesity; hypogonadotropic hypogonadism, hypocortisolism, elevated proinsulin and POMS concentrations in plasma but very low insulin level that indicates defective prohormone processing	Autosomal recessive	[56]

TUB	Member of the Tubby family of transcription factors	Participates in the control of the initiation of phagocytosis, promoting the removal of apoptotic cells by retinal pigment epithelial cells and macrophages	Early obesity with retinal dystrophy. Biallelic inactivating variants of TUB cause retinal abnormalities, defects in the basal body structure and morphology of the primary cilium axonemes, leading to defects in ciliogenesis	Autosomal recessive	[72]

CPE	Carboxypeptidase E	Participates in the biosynthesis of peptide hormones and neurotransmitters, including insulin	Morbid obesity, mental retardation, abnormal glucose homeostasis, and hypogonadotropic hypogonadism	Autosomal recessive	[73]

RAI1	Retinoic acid-inducible gene 1	A transcription factor, which is involved in cell growth and cell cycle regulation, bone and skeletal development, lipid and glucose metabolism, embryonic neurodevelopment and neuronal differentiation, behavioral functions, and circadian activity	Smith-Magenis-like obesity with hypoventilation, developmental disabilities	Sporadic, autosomal dominant	[74]

P4HTM	Member of the family of the hypoxia-inducible factor prolyl 4-hydroxylase (HIF-P4Hs)	Plays a key role in adaptation to conditions of insufficient oxygen supply	Severe obesity with hypotonia, cognitive impairment, and/or developmental retardation	Autosomal recessive	[75]

PHIP	Pleckstrin homology domain-interacting protein	Regulates the production of pro-opiomelanocortin (POMC). It is a probable regulator of insulin and insulin-like growth factor signaling pathways	Early childhood and adolescent obesity in children with mental and psychological disabilities	De novo, autosomal dominant with incomplete penetrance	[76]

NTRK2	Neurotrophic receptor tyrosine kinase 2	Regulates leptin-mediated synaptic plasticity of hypothalamic neurons	Obesity with cognitive impairment; impairments in memory, learning, and pain sensitivity, presumably due to altered hypothalamic function	Autosomal dominant	[77]
BDNF	Brain-derived neurotrophic factor ligand	Involved in energy balance and is an anorexigenic factor that is highly expressed in the ventromedial hypothalamic nuclei. BDNF function is regulated by MC4R and nutritional status		Autosomal dominant	[78]

## Data Availability

The data that support the findings of this study are available from the corresponding author upon reasonable request.
